# A common‐mesocosm experiment recreates sawgrass (*Cladium jamaicense*) phenotypes from Everglades marl prairies and peat marshes

**DOI:** 10.1002/ajb2.1411

**Published:** 2019-12-31

**Authors:** Jennifer H. Richards, Paulo C. Olivas

**Affiliations:** ^1^ Department of Biological Sciences Florida International University Miami Florida 33199 USA; ^2^ GIS/RS Center Florida International University Miami Florida 33199 USA

**Keywords:** A_max_, Cyperaceae, dauciform roots, soil and leaf N:P, nutrient deficiency, P‐limitation, sawgrass marsh, soil and leaf stoichiometry

## Abstract

**Premise:**

The southern Florida Everglades landscape sustains wetlands of national and international importance. Sawgrass (*Cladium jamaicense*), the dominant macrophyte in the Everglades, has two phenotypes that vary in size and density between Everglades marl prairies and peat marshes. Marl prairies have recently been hypothesized to be a newly formed habitat developed after European colonization as a result of landscape‐scale hydrologic modifications, implying that sawgrass marl phenotypes developed in response to the marl habitat. We examined whether sawgrass wetland phenotypes are plastic responses to marl and peat soils.

**Methods:**

In a common‐mesocosm experiment, seedlings from a single Everglades population were grown outdoors in field‐collected marl or peat soils. Growth and morphology of plants were measured over 14 mo, while soil and leaf total nitrogen, total phosphorus, total carbon, and plant biomass and biomass allocation were determined in a final harvest.

**Results:**

Sawgrass plant morphology diverged in marl vs. peat soils, and variations in morphology and density of mesocosm‐grown plants resembled differences seen in sawgrass plants growing in marl and peat habitats in Everglades wetlands. Additionally, sawgrass growing in marl made abundant dauciform roots, while dauciform root production of sawgrass growing in peat was correlated with soil total phosphorus.

**Conclusions:**

Sawgrass from a single population grown in marl or peat soils can mimic sawgrass phenotypes associated with marl vs. peat habitats. This plasticity is consistent with the hypothesis that Everglades marl prairies are relatively new habitats that support plant communities assembled after European colonization and subsequent landscape modifications.

Wetlands are major contributors to global climate regulation, storing amounts of carbon (C) equal to that found in forests and more than two‐thirds that found in the ocean (Moonmaw et al., 2018). Increased greenhouse gas concentrations in the atmosphere produce imbalances in the global C cycle, causing global climate changes, which in turn cause changes in vegetation distribution (Masson‐Delmotte et al., 2018; Nolan et al., 2018). Wetland plant communities, especially coastal wetland communities subject to sea‐level rise and saltwater intrusion, will respond to these environmental perturbations (Hopkinson et al., 2012; Short et al., 2016). How they respond will depend on the individual drivers and vegetation at affected sites. Experiments that dissect the type and magnitude of wetland vegetation changes are important to establish causes and predict outcomes of global climate changes (Franklin et al., 2016). Case studies for these responses provide (1) useful examples of the timing, type, and range of changes to be expected in response to wetland landscape modification; (2) ways to think about how changes impact the global C cycle; and (3) insights into restoring lost wetland productivity. Changes over the past century in wetland vegetation in the Everglades of southern Florida, USA, provide one such case study.

The Everglades sustain nationally and internationally recognized wetlands, including Everglades National Park (ENP), which is a World Heritage Site, an International Biosphere Reserve, and a RAMSAR Wetland of International Importance. Sawgrass (Cyperaceae: *Cladium jamaicense* Crantz) is the dominant macrophyte in the Everglades. This tall sedge reproduces vegetatively, as well as by seed, forming relatively monospecific populations that cover 50–80% of the landscape (Loveless, 1959; Davis et al., 1994; Richards et al., 2014). Over the past century, however, sawgrass abundance and distribution have changed, primarily as a result of anthropogenic modifications to the landscape (Davis et al., 1994; McVoy et al., 2011). Historically, sawgrass dominated a vast plain that extended south of Lake Okeechobee, Florida; much of this area has been converted to agricultural use. South of the plain, sawgrass occurred in elongated ridges that alternated with deeper water sloughs. Ridges and sloughs both paralleled the direction of flow. This patterned ridge‐and‐slough landscape occupied much of the Everglades south of the sawgrass plain (Davis et al., 1994; McVoy et al., 2011). Currently, sawgrass also occurs as a co‐dominant with muhly grass (*Muhlenbergia capillaris* [Lam.] Trin. [Poaceae]) in the marl prairies of the southern Everglades (Gunderson, 1994), where sawgrass plants are smaller and less dense (Appendix S1; see the Supplemental Data with this article). The marl prairies are among the most biodiverse habitats in the Everglades and provide habitat for a number of threatened and endangered species (Olmsted and Loope, 1984; Gunderson and Loftus, 1993).

Sawgrass marshes and marl prairies differ in soil type (peat vs. marl) and hydroperiod (long [≥8 mo] vs. short [<8 mo]), as well as location in the landscape (marl prairies peripheral, peat marshes central in ENP) (Olmsted et al., 1980; Olmsted and Loope, 1984; Gottlieb et al., 2006; Malone et al., 2014). These two communities also have different responses to climate change, especially as mediated by the El Niño Southern Oscillation (ENSO), that affect whether they are C sources or sinks (Moses et al., 2013; Malone et al., 2014) and thus may respond differently to future climate change, as well as to restoration.

A recent study of Everglades landscape history has questioned whether marl prairies were present before European settlement or are a novel landscape resulting from anthropogenic modifications (McVoy et al., 2011). McVoy et al. hypothesized that (1) the peripheral marl prairies were not present in the pre‐drainage Everglades; (2) current marl prairie areas were historically occupied by marl marshes that had a shallow layer of peat overlying marl; and (3) vegetation in the central and peripheral Everglades was thus much more similar, consisting primarily of sawgrass marshes. Under these hypotheses, current marl prairies developed as a result of drainage and burning in the first half of the 20^th^ century, which resulted in loss of the overlying peat. This anthropogenic landscape change produced large differences in the vegetation communities, as the present‐day marl prairies have a different species composition compared to sawgrass marsh (Olmsted and Loope, 1984; Gunderson, 1994; McVoy et al., 2011) and different productivity. The decrease in sawgrass size and density in marl habitats, as well as the loss of peat soil, also decreased C storage. If these hypotheses are true, they provide insight into how rapidly the Everglades landscape can change with changing climate and hydrology, as well as an increased understanding of the historical contribution of this landscape to the global C cycle and how it might change with restoration.

Peat soils are common in the Everglades (Obeysekera et al., 1999; McVoy et al., 2011). They have low bulk density and high percent organic matter (>60%; Stober et al., 1998). Peat soils vary in total nitrogen (TN) and total phosphorus (TP) levels at different sites in the Everglades (Craft and Richardson, 1993a, b; Newman et al., 1997). Marl soils, which are freshwater calcitic muds, are currently found as surface soils on the eastern and western edges of the southern Everglades (Obeysekera et al., 1999; McVoy et al., 2011). These highly inorganic soils are derived from freshwater, calcium‐carbonate‐precipitating cyanobacteria that form benthic, epiphytic, or floating mats in shallow freshwater marshes. Marls have very high bulk density, low percent organic matter (<40%; Stober et al., 1998), and low TN and TP.

Sawgrass phenotypes in peat habitat are tall (>2 m) and densely distributed on deeper peat (>1 m deep) or shorter (80–150 cm) with intermediate densities on shallower peat (Gunderson, 1994). Sawgrass plants in marl prairies are typically short (<1 m) and sparsely distributed (Gunderson, 1994; Appendix S1). If the marl prairie was present in the pre‐drainage Everglades, sawgrass growing there might be an ecotype distinct from sawgrass growing on the sawgrass plain and ridges. Whether these different sawgrass morphologies are ecotypes or are plastic responses to the different soil types, hydrology, or other environmental conditions found in peat and marl habitats is not known.

The Everglades is an oligotrophic wetland in which P is the limiting macronutrient (Noe and Childers, 2007). Plants can produce different root morphologies, such as dauciform roots, that enhance P uptake (Lambers et al., 2006; Raven et al., 2018). Dauciform roots are short lateral roots with abundant root hairs that excrete carboxylates and phosphatases; these can solubilize sorbed P, increasing P availability in inorganic, nutrient‐poor soils, thus providing an ecophysiological advantage in P acquisition (Shane et al., 2005, 2006; Playsted et al., 2006). *Cladium* species have been reported to produce dauciform roots (Davies et al., 1973; Lamont, 1982), and we have observed such roots on sawgrass (J. H. Richards, personal observation). Since nutrient availability differs between peat and marl soils, the production of this type of root could also differ in these two environments, with more dauciform roots in soils with less P. There are no reports, however, of variation in sawgrass dauciform root production in the field, and whether dauciform root production varies in marl vs. peat soils is not known.

The purpose of this study was to examine whether sawgrass from a single source could reproduce marl and peat sawgrass phenotypes. We used sawgrass seeds collected from a single population and grown in marl or peat soil to see if they would mimic the variation in sawgrass found across the Everglades landscape. We examined growth; shoot and root morphology, including dauciform root abundance; biomass allocation; soil and plant nutrient status; and photosynthetic rates. We hypothesized that the morphological differences in sawgrass recognized in field populations (short, sparse plants in marl vs. tall, dense plants in peat; Gunderson, 1994) are plastic responses to differences in soil type.

## MATERIALS AND METHODS

### Seed source and growing conditions

Seeds were collected from multiple individuals in the Hole‐in‐the‐Donut RES2004E restoration block, ENP (northeast corner = 25°22′30″N, 80°37′19″W), in fall 2010. This site, originally covered by an invasive exotic shrub, was scraped to bedrock in 2004 and allowed to regenerate naturally from seeds dispersed from the surrounding area. Thus, sawgrass at the site was established by seed, then plants enlarged through vegetative reproduction over the 6 yr prior to seed collection. The bulked seeds were planted in potting soil in trays with insets, several seeds per inset, at the Daniel Beard Research Center greenhouse at ENP in February 2011. Seeds germinated and grew over the next 3 mo, then were brought to Florida International University's Modesto Maidique campus (FIU_MMc). On 23 May 2011, seedlings were transplanted individually into 20 × 12.5 cm plastic pots filled with either peat or marl soil. The peat soil was collected in the Florida Everglades in Water Conservation Area (WCA) 3A or in the South Florida Water Management District's Storm Water Treatment Areas. These soils, which came from multiple locations, were not homogenized prior to use, and individual pots had different soil origins. The marl soil was native marl collected from the Cemex USA quarry on Card Sound Road in Florida (25°24′14″N, 80°28′5″W).

The 83 planted seedlings (42 in peat and 41 in marl) were allowed to acclimate in shallow pools for 10 d, then morphological measurements were taken. At this time, seedlings in the two soil types did not differ from each other in length of the most recently matured leaf (peat = 8.4 ± 2.9 cm; marl = 8.7 ± 3.2 cm, *N* = 83, Kruskal‐Wallis [KW] χ^2^ = 0.28, *P* = 0.59). The number of live leaves, while similar (median = 3, range: 1–5 for both soil types, *N* = 83), was slightly less in marl (KW χ^2^ = 6.33, *P* = 0.01). The transplants were placed on shelves in two water‐filled mesocosms (3415 L) set outside at FIU_MMc; plants in peat alternated with plants in marl along the mesocosm shelves. During the experiment, water level varied from several centimeters above the pot soil surface to several centimeters below, but the pots were always sitting in water.

On 2 July, 2012, triplicate water samples were taken from each mesocosm to determine total dissolved N and P, while soil cores were taken from a randomly selected subset of the peat (*n* = 30) and marl (*n* = 31) samples. Bulk density, pH, and soil TN, TP, and total carbon (TC) were determined from these samples. Water and soil analyses followed methods described in Serna et al. (2013) and were based on EPA method 365.1 (US EPA, 1983), Nelson and Sommers (1982), and Solórzano and Sharp (1980). Air temperature for the duration of the study was downloaded from a weather station at FIU_MMc maintained by Dr. Rene Price (https://www.wunderground.com/history/monthly/us/fl/miami/KMIA/date/2019-8?cm_ven=localwx_history). Data for water nutrients and air temperature are provided in Appendix S2.

### Growth and photosynthesis measurements

Growth of plants was monitored at 1 mo intervals in the active growing season or 2 mo intervals in the slower growing season (November–March). Length and width of the most recently matured leaf were measured, and this leaf was marked with indelible ink. The number of new leaves that had expanded since the last sample, the number of live leaves, and the number of branches on the main stem were counted. Whether flowering had occured or the original apex had died was recorded. If the original apex died, the most vigorous branch was measured and marked as a replacement. The status of the original shoot (alive or dead) was censused at the June 2012 sampling.

Photosynthetic rate was measured on a subset of 10 plants from each soil type on 3–4 April 2012, and on 10 July 2012; nine plants of the sampled plants (6 in peat, 3 in marl) were measured on both dates. We measured the photosynthetic capacity at saturating light conditions (A_max_) with an infrared gas analyzer (LI‐COR 6400, Lincoln, Nebraska, USA) using the internal light source. The intensity was set at 1200 μmol m^−2^ s^−1^. Internal CO_2_ was set at 400 ppm, temperature at 25°C, and moisture at 85%. Before measurements were taken, the leaf inside the chamber was acclimatized to 1200 μmol m^−2^ s^−1^ of light until a steady photosynthetic rate was observed, then A_max_ was recorded. Measurements were made between 0900 and 1500 hours on sunny days. Data collected were A_max_ (maximum photosynthetic capacity) and stomatal conductance (rate of exchange of CO_2_ or water vapor through the stomata).

### Biomass and tissue nutrient determination

To determine biomass and biomass allocation to plant parts, 61 plants (30 growing in peat, 31 in marl) were harvested from 11 through 27 July 2012. Each plant was photographed, removed from its pot, and the soil was washed from leaves, stems, and roots. Washed plants were separated into roots, stems, and live and dead leaves, dried at 70°C, and weighed to the nearest 0.01 g. A subsample of healthy, mature leaf tissue from each plant was analyzed for TC, TN, and TP following the methods described for soil nutrient analyses. These data were used to compute C:N, C:P, and N:P mass ratios.

### Dauciform root analysis

During the final harvest, a single soil core was taken from each of 29 plants (14 growing in peat, 15 in marl) in order to assay dauciform root presence and abundance. The cores were 2.4 cm in diameter, and core length averaged 6.0 cm, for an average volume of 27 cm^3^ per core. Cores were gently washed with tap water and then examined for dauciform root presence and relative abundance using a dissecting microscope equipped with a digital camera. Samples were dried in an oven at 70°C and weighed to the nearest 0.001 g. Each sample's biomass was added to the total root biomass for the plant from which it came.

Dauciform root relative abundance was quantified using a dauciform root index (DRI) to estimate the relative abundance of these roots in each core. The DRI ranged from 1 to 10, with each integer representing a 10% interval, beginning with 1, which indicated that 0–10% of the roots viewed under the dissecting microscope were dauciform roots.

### Statistical analyses

Variances of most of the biomass and nutrient variables were unequal when peat and marl soil samples were compared with Levene's test for homogeneity of variances. Theses variables were therefore compared using nonparametric Kruskal‐Wallis (KW) tests. Similarly, the dauciform root indices were analyzed with KW tests. Differences in percent biomass allocation were analyzed using regression analysis of percent allocated to one organ type or another in each soil type; slopes of the regression lines were compared with analysis of covariance. Model simplification was used to compare regressions of biomass to soil nutrients with interactions vs. no interactions using the Akaike information criterion (AIC); models with lower AIC were considered to be better. Photosynthetic rates on different dates and in different soils were analyzed with analysis of variance. Statistical analyses and data visualizations were done in the R statistical environment (R Core Team, 2017). In addition to the base package, we used ggplot2 (Wickham, 2016), plyr (Wickham, 2011), PMCMR (Pohlert, 2016), and nlme (Pinheiro et al., 2018).

## RESULTS

### Soil characteristics

**Figure 1:**
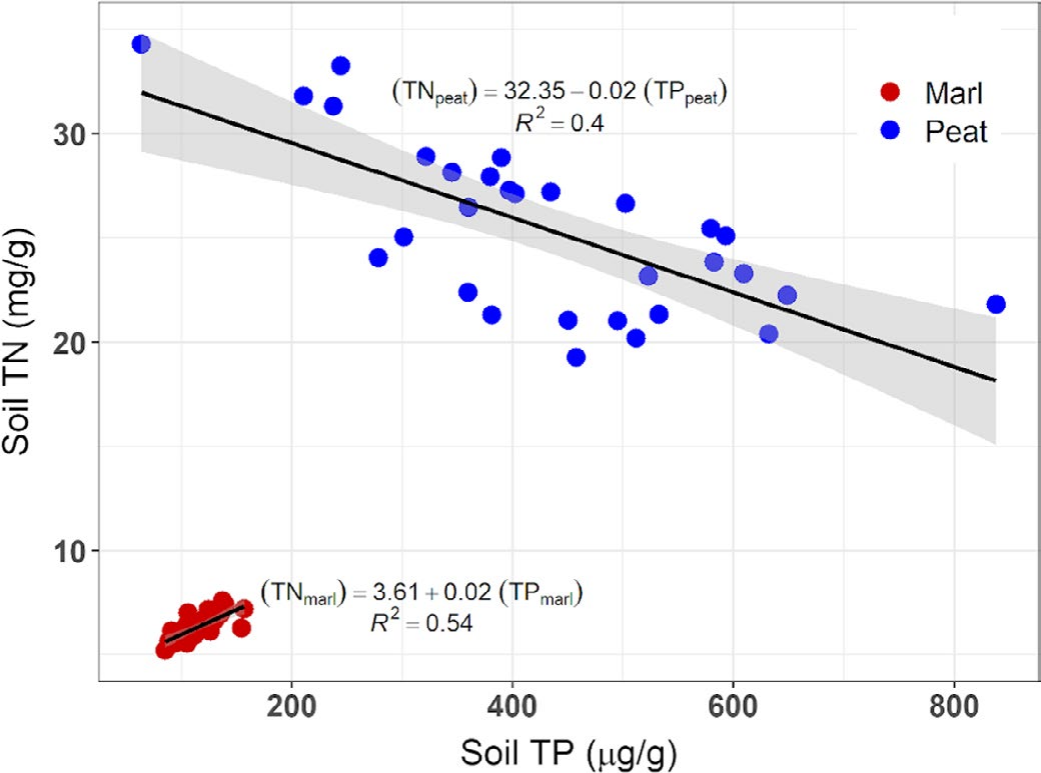
Correlation of total nitrogen (TN) vs. total phosphorus (TP) in marl and peat soils used in the *Cladium jamaicense* mesocosm experiment. The slopes of linear models for TN by TP have different directions in peat vs. marl soil.

Physical characteristics and nutrients varied significantly between peat and marl soils (Table 1). Marl soil had a greater field bulk density and higher pH than peat, but lower TN, TP, and TC. Peat soils had much greater variation in nutrient levels than marl (Table 1; Fig. 1). Peat soils had greater TP and TN than marl (Fig. 1). In addition, TN and TP were correlated in both peat (*F*_1,27_ = 17.9, *P* = 0.00) and marl (*F*_1,29_ = 34.2, *P* < 0.00) but varied inversely (Fig. 1). Despite these differences, N:P was not significantly different between the soil types and was high in both (55_marl_, 61_peat_; Table 1).

**Table 1:** Mean values (± SE) of marl and peat soil variables for soils used in *Cladium jamaicense* (sawgrass) mesocosm experiments and for sawgrass plants grown in those soils (*n* = 31 grown in marl, *n* = 30 grown in peat). Nutrient ratios are mass ratios.

Variable	Marl	Peat	Prob.	P:M
FBD_soil_ (g DM/cm^3^)	0.55 ± 0.01	0.26 ± 0.01	<0.01	0.47
pH_soil_	7.57 ± 0.02	7.35 ± 0.02	<0.01	0.97
TN_soil_ (mg/g DM)	6.38 ± 0.11	24.78 ± 0.67	<0.01	3.88
TN_plant_ (mg/g DM)	8.16 ± 0.14	7.12 ± 0.16	<0.01	0.87
TC_soil_ (mg/g DM)	153.74 ± 0.75	396.63 ± 8.93	<0.01	2.58
TC_plant_ (mg/g DM)	447.41 ± 0.79	444.08 ± 0.93	0.01	0.99
TP_soil_ (μg/g DM)	116.85 ± 3.3	456.83 ± 26.33	<0.01	3.91
TP_plant_ (μg/g DM)	218.59 ± 5.65	285.92 ± 24.84	0.14	1.31
N:P_soil_	55.3 ± 1.0	61.1 ± 5.2	0.99	1.10
N:P_plant_	37.9 ± 1.0	28.9 ± 2.0	<0.01	0.76
C:N_soil_	24.3 ± 0.3	16.1 ± 0.1	<0.01	0.66
C:N_plant_	55.3 ± 1.0	63.1 ± 1.2	<0.01	1.14
C:P_soil_	1344 ± 34	970 ± 74	<0.01	0.72
C:P_plant_	2085 ± 50	1821 ± 123	0.13	0.87

Notes

FBD = field bulk density; DM = dry mass; TN = total nitrogen; TC = total carbon; TP = total phosphorus; Prob. = probability of a greater χ^2^ value in a Kruskal‐Wallis test; P:M = ratio of peat to marl.

### Plant growth responses

**Figure 2:**
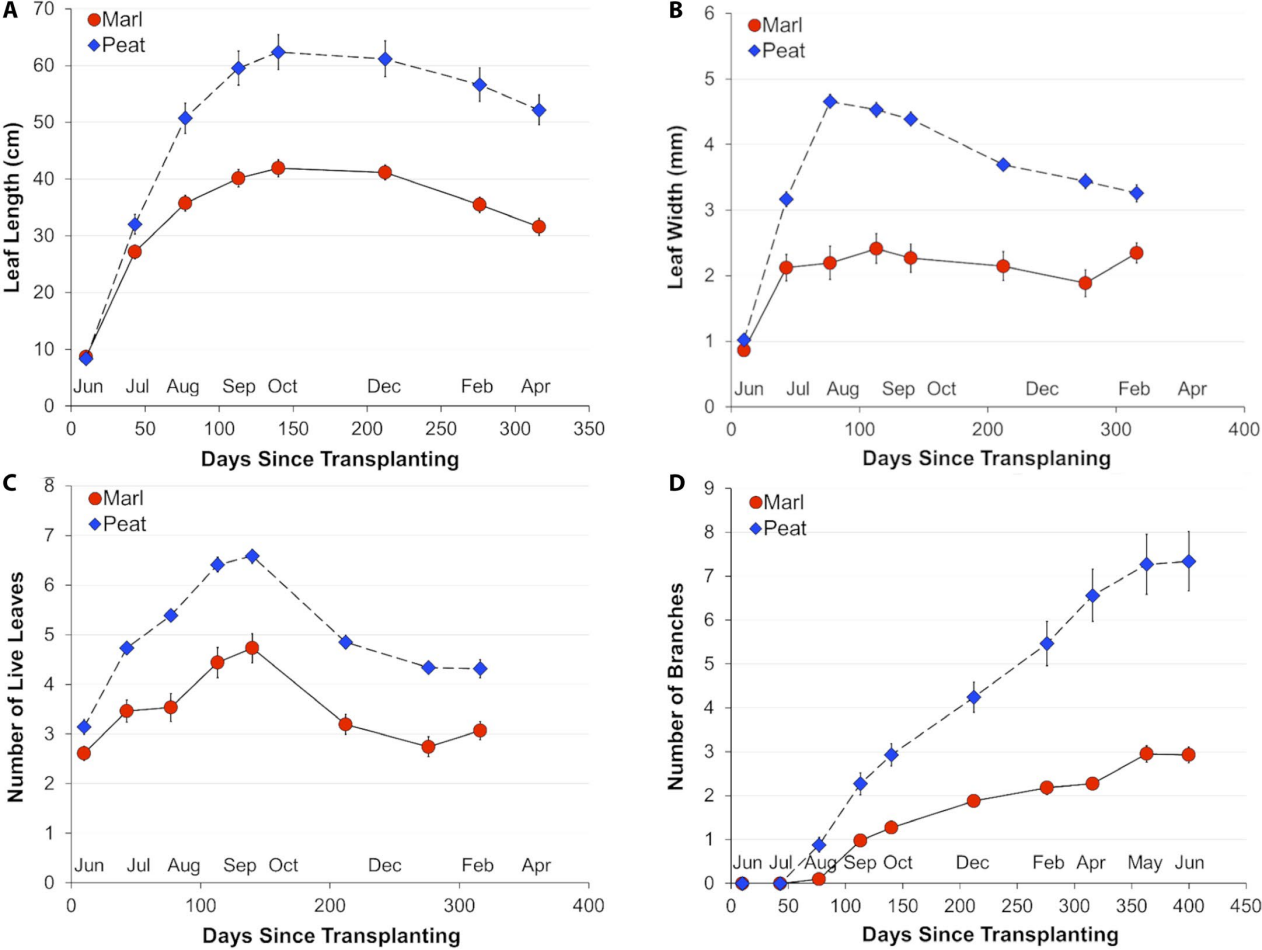
Growth of *Cladium jamaicense* over time in marl and peat soils: (A–C) data for 10.5 mo; (D) data for 14 mo.

The growth of seedlings in peat began to exceed that of seedlings in marl within the first month of treatment (Fig. 2). Leaf length (Fig. 2A) increased on seedlings in both peat and marl from initial measurements in June–September 2011, then stabilized in both soil types in the cooler winter months (Fig. 2; Appendix S3). Leaf length began to decline in the spring months, coincident with shoot death in many of the original shoots. Leaf width increased in the first month in both soil types and continued to increase on plants in peat but not marl (Fig. 2B). The number of live leaves also increased from June through September/October, then declined (Fig. 2C). Although seasonal patterns of growth were similar in seedlings in the two soils, both leaf length and the number of live leaves on plants were greater in peat than in marl (e.g., by 49% and 39%, respectively, in October). Plants in peat began to branch after 2 mo of growth, and the number of branches in peat increased steadily until May (Fig. 2D). Branching onset lagged on plants in marl, and these plants branched less (2.3_marl_, 6.6_peat_; Appendix S3). The number of branches in both soil types increased through the year, but from October through April the increase in marl was much less than that in peat (Fig. 2D). Branch number varied more among plants in peat than among those in marl (SE = 0.6_peat_ vs. 0.1_marl_; Appendix S3). The cumulative effect of differences in growth was that plants in peat had longer, wider leaves, more live leaves per culm, and more branches than plants in peat (Fig. 2; Appendix S3).

The original shoot aborted over time in both soil types, but more shoots aborted in marl than in peat. By May 2012, the original shoot of plants in marl had died on 46% of the plants, another 34% were dying, and only 19% were continuing to grow. In the May 2012 sample of plants in peat, only 17% of the original shoots had died, 33% were dying, and 50% were continuing to grow. Two plants growing in peat flowered, whereas none flowered in marl.

All the morphological variables were significantly different between soil types in April after 10.5 mo of growth in the mesocosms (Appendix S3). After 14 mo of growth, however, these differences had disappeared for all variables except branch number, probably because the main shoot apex in many plants died and the ensuing switch to monitoring the largest branch led to increased variation in measurements among plants, as the branches produced smaller leaves.

### Photosynthesis

**Figure 3:**
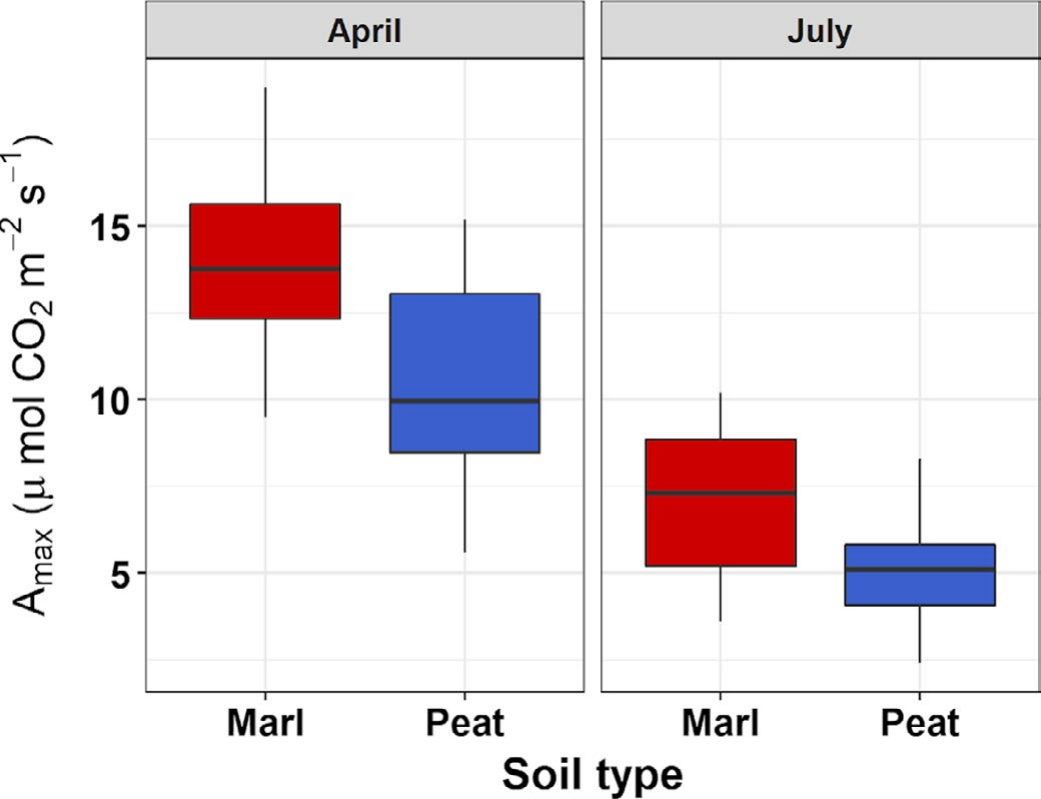
Maximum photosynthetic capacity (A_max_) for *Cladium jamaicense* growing in marl and peat soils in mesocosms in April and July 2012.

Leaf A_max_ differed between plants growing in marl and peat soils (Fig. 3). There were only weak relationships between photosynthetic parameters for the two dates (*R* ^2^ = 0.06 for A_max_, *R* ^2^ = 0.14 for stomatal conductance; *n* = 9 plants measured on both dates). When data were modeled for effects of substrate, date, and mesocosm on A_max_, substrate and date were highly significant (*F*_1, 36_ > 12.15, *P* < 0.00), but mesocosm was not (*F*_1, 36_ = 3.51, *P* = 0.07). Plants in marl had a significantly greater A_max_ than those in peat, and A_max_ was greater in the spring than in the summer (Fig. 3). If data were examined by date, A_max_ differed significantly with substrate in April (*F*_1, 18_ = 7.15, *P* = 0.02); but in July, differences were only marginally significant (*F*_1, 18_ = 4.24, *P* = 0.05).

Plant nutrients were analyzed for plants harvested soon after the July photosynthesis measurements were taken. When A_max_ from July was modeled as a function of plant TN and TP, A_max_ increased with leaf TN (*F*_3, 16_ = 5.02, *P* = 0.04) but not with leaf TP (*F*_3, 16_ = 1.23, *P* = 0.28), while the TN*TP interaction was marginally significant (*F*_3, 16_ = 3.50, *P* = 0.08). The overall model was significant (*F*_3, 16_ = 3.25, *P* = 0.05), and *R* ^2^_adj_ = 0.26. If the TN*TP interaction was removed from the model, the model lost its significance (*F*_2, 167_ = 2.72, *P* = 0.09).

When stomatal conductance was modeled with substrate, date, and mesocosm, the model was not significant (*F*_5, 32_ = 0.69, *P* = 0.69). The residuals were not normally distributed when two outliers were present. Analysis of the effects of substrate alone on stomatal conductance was not significant with the outliers removed.

### Plant biomass, nutrient content, and dauciform root abundance

Biomass of leaves (live, dead, and total leaf biomass), stems, roots, and total biomass of plants in peat was significantly greater than that of plants in marl (Table 2). Biomass allocation to different plant parts also differed significantly between soil types, except for the proportion of stem and the ratio of live to dead leaf biomass (Table 2). Plants in peat had 4× or more the biomass of plants in marl (Table 2). Despite the differences in biomass, plants in marl and peat had similar scaling relations for different plant parts. In a comparison of regression models for ln biomass in leaves or roots, slopes were similar in peat vs. marl soils (AIC for a model without interactions = 28.49; AIC for a model with interactions = 28.07). The simpler model without interactions (= similar slopes) was accepted.

**Table 2:** Mean biomass (± SE) and proportion of total biomass for different plant parts harvested from *Cladium jamaicense* planted in saturated Everglades marl or peat soils and grown for 14 mo (*n* = 31 grown in marl, *n* = 30 grown in peat).

Plant part	Marl	Peat	Prob.	P:M
Live leaves (g)	3.53 ± 0.28	14.93 ± 2.03	<0.01	4.23
Dead leaves (g)	1.08 ± 0.13	4.53 ± 0.58	<0.01	4.19
Live:dead leaves	3.92 ± 0.36	3.43 ± 0.28	0.20	0.84
Total leaves (g)	4.61 ± 0.40	19.46 ± 2.58	<0.01	4.22
Stem (g)	1.03 ± 0.12	4.66 ± 0.68	<0.01	4.52
Total live shoot (g)	4.56 ± 0.38	19.59 ± 2.69	<0.01	4.30
Total shoot (g)	5.64 ± 0.50	25.70 ± 3.23	<0.01	4.28
Total root (g)	4.29 ± 0.38	21.45 ± 3.27	<0.01	5.00
Total live (g)	8.85 ± 0.72	41.04 ± 5.87	<0.01	4.64
Total plant (g)	9.94 ± 0.83	45.57 ± 6.39	<0.01	4.58
DR core (mg)	30.3 ± 7.9	62.8 ± 6.4	<0.01	2.07
Proportion live leaves	0.36 ± 0.01	0.32 ± 0.01	0.01	0.89
Proportion leaves	0.47 ± 0.01	0.43 ± 0.01	0.04	0.91
Proportion roots	0.43 ± 0.01	0.47 ± 0.01	0.04	1.09
Proportion stem	0.10 ± 0.01	0.10 ± 0.00	0.48	1.00
Shoot:root	1.39 ± 0.07	1.21 ± 0.07	0.06	0.87

Notes

Prob. = probability of a greater χ^2^ value in a Kruskal‐Wallis test; P:M = ratio of peat to marl; DR = dauciform root.

Sawgrass plants in marl and peat soils differed significantly in leaf TN and TC (Table 1). Leaves on plants in marl had greater TN and TC than leaves on plants in peat. The TP did not differ significantly between soil types, probably because of the high variation in TP for peat (Table 1). Mass ratios of N:P and C:N were significantly different between plants in the two soil types, although C:P ratios were not (Table 1). Although peat had much greater TN than marl, leaf TN of all plants was more similar to TN levels in marl, and plants in marl had greater TN than plants in peat (Table 1). Similarly, although peat had much greater TP than marl, plants in the two soils did not differ in TP (Table 1). Plants in marl had significantly higher N:P ratios than plants in peat (Table 1), suggesting greater P limitation for plants in marl.

When soil nutrients (TN, TP, TC) were used to predict total plant biomass, the best model (lowest AIC) had only soil TP (AIC = 550). This model was better than a model with TN or TC alone (AIC = 581 and 580, respectively) or with TN and TC together (AIC = 579).

**Figure 4:**
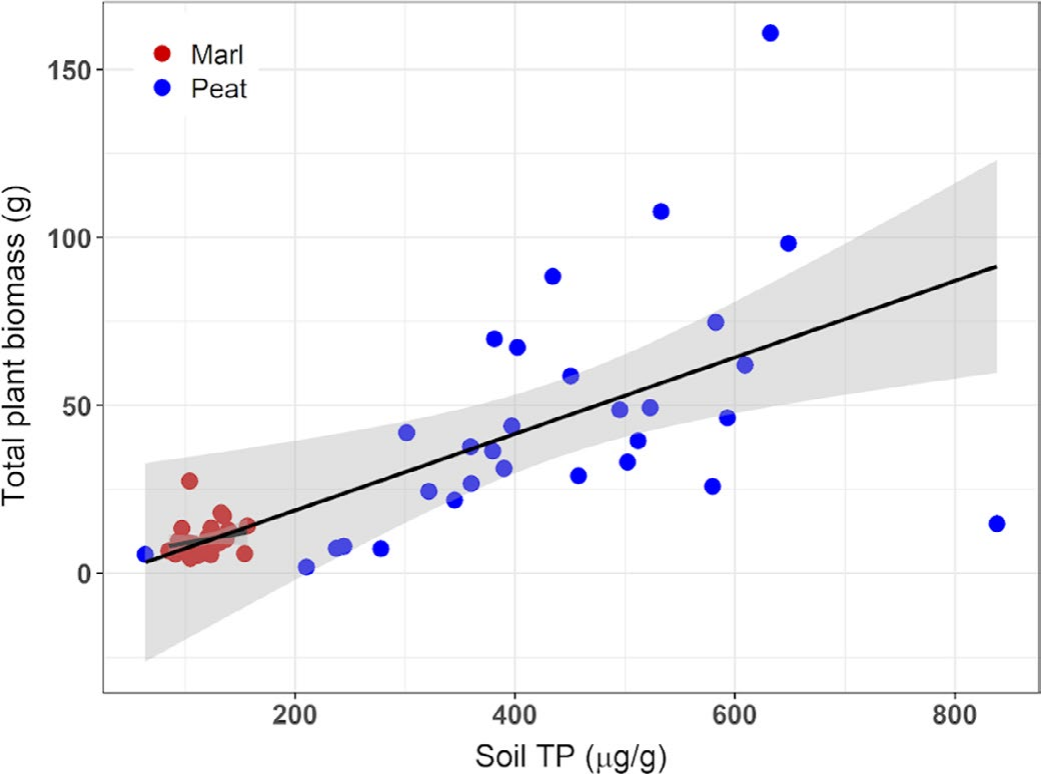
The correlation of total phosphorus (TP) in the soil to total plant biomass for *Cladium jamaicense* growing in peat and marl soils. Linear models for biomass vs. soil TP in marl and peat do not have significantly different slopes.

Total biomass scaled similarly with soil TP in both soil types. A comparison of the slopes for total biomass vs. soil TP showed no significant difference in the slope of the two (AIC for a model with interactions = 554; AIC for a model without interactions = 552) (Fig. 4).

**Figure 5:**
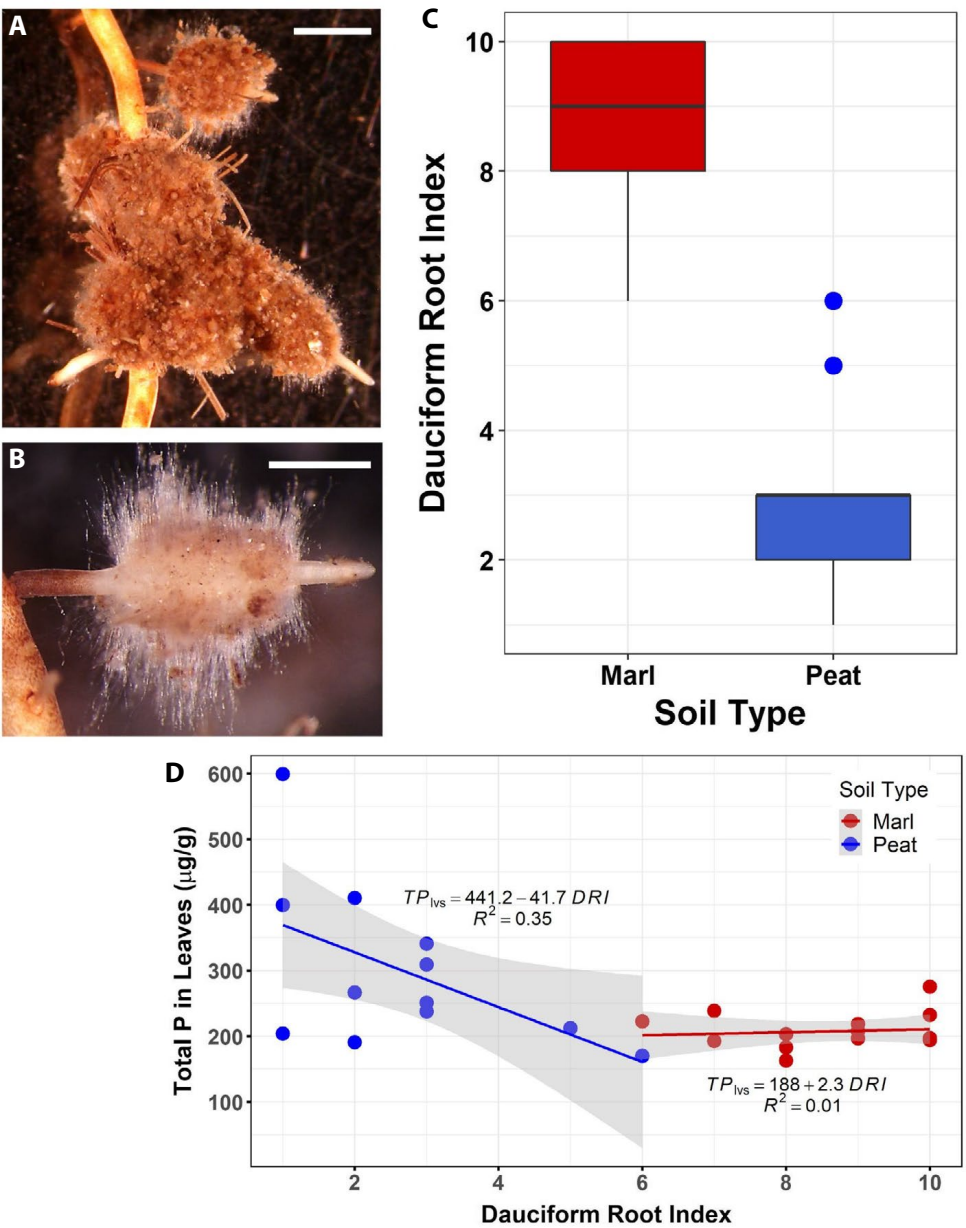
Dauciform roots in *Cladium jamaicense* (sawgrass). (A) Several lateral dauciform roots growing from a sawgrass root, with some soil remaining trapped in long, dense root hairs of dauciform roots (scale bar = 2 mm). (B) A single lateral dauciform root with soil removed, exposing the long, dense, dauciform root hairs coming off the swollen part of the root (the root's base is on the left and its apex is on the right; scale bar = 1 mm). (C) Dauciform root index (DRI), which reflects the relative abundance of dauciform roots, for root samples from plants growing in different soil types (marl vs. peat). (D) DRI in relation to total phosphorus (TP) in leaves of plants in marl and peat.

Plants in both soil types produced lateral dauciform roots that trapped soil particles in elongated root hairs (Fig. 5A, B). Although the root biomass in marl cores was approximately one‐half that in peat, plants in marl had a greater dauciform root index (DRI) than plants in peat (DRI = 9_marl_ vs. 3_peat_; Fig. 5C) and thus ~60% more dauciform roots (KW χ^2^ = 20.07, df = 1, *P* < 0.00). For plants in peat, the TP in leaves varied, and the DRI varied inversely with leaf TP (Fig. 5D). Plants in marl had higher DRIs with less variation (Fig. 5D).

## DISCUSSION

### Sawgrass morphological responses to marl and peat

Sawgrass plants grown from seed collected from a single population but transplanted into marl or peat soil and grown together in mesocosms differed in growth and morphology between the soil types. If the sawgrass phenotypes described in the field were ecotypes, seeds from a single population should have a narrow range of phenotypes, and plants in peat should have resembled those in marl. Instead, the variations seen among plants in peat and between plants in marl and peat were correlated to soil TP. In addition, the range in sawgrass morphology seen across the Everglades ecosystem was reflected in the mesocosm plants and paralleled the differences in sawgrass found in peat vs. marl habitats in the field (Gunderson, 1994). Thus, our experimental results support the hypothesis that the morphological differences in sawgrass recognized in field populations (short, sparse plants vs. tall, dense plants; Gunderson, 1994) are plastic responses to differences in soil type.

### Influences of soil nutrients on sawgrass phenotypic differences

The marl and peat soils used in this experiment had nutrient concentrations similar to marl and peat soils found in Everglades field samples (for marl, see Osborne et al., 2011; Liao and Inglett, 2012; Sah et al., 2014; for peat, see Craft and Richardson, 1993a, b; Newman et al., 1997; Osborne et al., 2011). The sawgrass morphological and biomass differences in our experiment are associated with differences in soil TP but not soil TN. Studies of variation in sawgrass morphology in peat soils that differ in TP have also shown that sawgrass biomass increases with increasing soil TP (Miao, 2004; Weisner and Miao, 2004) or with TP, but not with TN addition (Craft and Richardson, 1997). Sawgrass grown in hydroponic solutions with varying TP levels also increased in biomass with increasing TP (Lorenzen et al., 2001; Lissner et al., 2003; Webb and Zhang, 2013). Branching increased with increased TP, either along nutrient gradients or with added TP (Steward and Ornes, 1983; Brewer, 1996; Miao et al., 1997; Lorenzen et al., 2001; Lissner et al., 2003; Miao, 2004). Webb and Zhang (2013) found no branching over a TP range, but they worked with plants <6 mo old that may not have had the ability to branch; in our experiment, plants did not branch until the seedlings were ≥6 mo old.

Sawgrass growth thus reflects a feedback loop between the plant, soil, and hydrology. Both hydrology and sawgrass growth are drivers in this loop. In peat, sawgrass growth builds more peat by adding organic biomass to the soil (Obeysekera et al., 1999). In areas with longer hydroperiods with short or no dry‐downs, decomposition of peat through oxidation of organic matter is reduced and peat accumulates over time, further enhancing sawgrass growth and forming the thick deposits characteristic of pre‐drainage Everglades marshes (McVoy et al., 2011). In marl areas, sawgrass plants have less growth and thus less production of organic matter, so peat accumulates more slowly. The shorter hydroperiod at marl sites allows organic matter to oxidize when sites are dry, preventing peat buildup and maintaining slow sawgrass growth. Restoration of Everglades marshes thus requires restoration of longer hydroperiods but will also need time to establish enough peat to support enhanced sawgrass growth.

Plants in the two soil types scaled biomass allocation to different plant parts similarly, but plants in peat grew more, producing at least 4× more roots, stems, and leaves. This lack of change in biomass allocation has been noted in experimental studies of sawgrass response to different levels of P (Miao et al., 1997; Lorenzen et al., 2001; Miao, 2004), although Lissner et al. (2003) found a 2.5× greater shoot:root ratio at 500 μg P L^−1^ vs. 10 μg P L^−1^ in hydroponics.

Plant N:P mass ratios >16 generally indicate P‐limitation, ratios <14 indicate N‐limitation, and ratios between 14 and 16 can indicate either N‐ or P‐limitation or co‐limitation (Koerselman and Meuleman, 1996; Bedford et al., 1999; Güsewell and Koerselman, 2002; Güsewell, 2004). Leaf N:P ratios for our plants thus suggest severe P‐limitation in both soil types, with marl soils (N:P = 38 ± 1) being more severely P‐limited than peat soils (N:P = 28 ± 2). In a meta‐analysis of wetland nutrient studies, Güsewell and Koerselman (2002) reported an N:P range of 3–40 among 1248 samples from European wetland species, with only four values >40 from two out of 84 species. In that study, *Cladium mariscus*, a European congener of *C. jamaicense*, had a mean N:P of ~16 (Güsewell and Koerselman, 2002). The N:P ratios reported for sawgrass in the Everglades are from peat‐based systems or for plants grown in hydroponics. These N:P ratios vary with TP concentration in the soil or nutrient solutions. Plants in undisturbed peat typically have P‐limited values (28 in ENP [Daoust and Childers, 1999], 29–31 in WCA 2B [Chiang et al., 2000], 34–38 in WCA 2A [Richardson et al., 1999]). Plants in enriched peat or peat fertilized with P have N‐limited or N and P co‐limited values (14–16 in WCA 2B [Chiang et al., 2000], 11–16 in WCA 2A [Richardson et al., 1999]). The N:P ratio for sawgrass in hydroponic solutions with 10 μg P L^−1^ was 32–37, while the ratio in 80 or 500 μg P L^−1^ was 14–24 (Lorenzen et al., 2001).

### Sawgrass photosynthesis in marl and peat soils

The higher A_max_ found in sawgrass plants in marl soil was counterintuitive, given that the plants in marl were smaller and had much lower biomass. A_max_ typically increases with increasing leaf TN in both natural and artificial conditions (Baddeley et al., 1994; Peng et al., 1995; Ripullone et al., 2003; Wright et al., 2004; Boussadia et al., 2010). Our plants in marl had greater leaf TN than our plants in peat, despite the marl soil having much lower TN (Table 1). Reich et al. (2009) found, in a survey of 314 species distributed from boreal to tropical habitats, that in systems that are P‐limited, the A_max_ to N relationship may be constrained by low P, as the reduced amount of P may inhibit ribulose‐1,5‐bisphospate regeneration in the dark reactions of photosynthesis (Warren and Adams, 2002). The positive correlation of A_max_ to leaf TN combined with the lack of correlation between A_max_ and leaf TP, but the presence of a significant interaction of leaf TN*TP suggests that such a TP constraint is operating on photosynthesis in plants in marl soil. Low supplies of N and P, however, usually limit plant growth primarily by restricting leaf growth and secondarily by decreasing photosynthetic rate (A_max_; Reich et al., 2009). In our experiment, leaf size and branch production were reduced in marl compared to peat. This general growth reduction was probably responsible for the decreased biomass, despite increased A_max_ for plants in marl. The fact that biomass increase was positively correlated with TP for plants in all soils supports the interpretation that reduced soil nutrients cause the major biomass differences between plants in marl and peat. In a field fertilization experiment on peat soils (WCA 2B), control, high P, and high N treatments did not differ in A_max_ (13, 12, and 13 μmol m^−2^ s^−1^), whereas plants in high P + high N had higher A_max_ (15 μmol m^−2^ s^−1^) (Chiang et al., 2000). Juvenile plants that were pulsed with nutrients (both N and P) had higher A_max_ (12 μmol m^−2^ s^−1^) than control plants (10 μmol m^−2^ s^−1^) (Miao et al., 1997). Hydroponic experiments with 10 μmol P or 100 μmol P found no significant difference in CO_2_ assimilation rate (A_max_ = 9.3 in 10 μM P; A_max_ = 10.5 in 100 μM P). Lorenzen et al. (2001), however, found a lower A_max_ in plants grown in 10 μg P L^−1^ (A_max_ = 1–4 μmol m^−2^ s^−1^) than in plants grown in 80 or 500 μg P L^−1^ (A_max_ = 10–14 μmol m^−2^ s^−1^).

### Dauciform roots in sawgrass

Arbuscular mycorrhizae, which are common fungal–root symbioses that enhance nutrient uptake, are less common in wetland plants, especially wetland monocots such as sawgrass (Fusconi and Mucciarelli, 2018). Plants in stressful environments frequently have some other morphological and/or physiological modification than mycorrhizae to enhance nutrient uptake (Brundrett, 2009; Raven et al., 2018). In the present study, sawgrass plants produced dauciform roots, an alternative root modification found in some sedges (Lamont, 1982; Shane et al., 2005). Dauciform roots were present on plants in both peat and marl soil, but more dauciform roots were produced in marl. Experimental hydroponic studies that manipulated P levels have demonstrated that dauciform root production in other sedge species is a plastic response to P availability, with more dauciform roots produced in lower P (Shane et al., 2005, 2006; Playsted et al., 2006). The same correlation was seen in our experiment for plants in peat soils over a range of TP concentrations. This response was not present, however, at the very low TP levels in marl, where dauciform root production was high in all samples. Thus, dauciform root production is plastic in response to soil TP; but at very low levels of TP (high nutrient stress), plants reach a maximum level of dauciform root production. Studies of sawgrass growth in response to soil type or nutrients in southern Florida have not described dauciform roots, although Lissner et al. (2003) mentioned the production of cluster roots only in their 10 μg P L^−1^ hydroponic treatment, not in the 80 or 500 μg P L^−1^ treatments; these cluster roots were probably dauciform roots. Although Webb and Zhang (2013) did not find dauciform roots on juvenile sawgrass plants grown on solid media, they did find increased acid phosphatase activity in the roots of plants in low P (10 μmol L^−1^) as opposed to high P (100 μmol L^−1^), as well as longer roots and more lateral roots in low P. Dauciform roots are lateral roots that secrete acid phosphatase, so this physiological capacity in the juvenile plants used by Webb and Zhang (2013) may presage the production of dauciform roots in older plants.

### Implications of sawgrass responses for Everglades restoration and global climate change

Today's extensive marl prairies in the Everglades ecosystem may be a novel habitat created in response to anthropogenic modifications over the past century, as suggested by McVoy et al. (2011). Sawgrass plants in the marl prairies have one‐fifth the biomass of plants in peat, so this sawgrass has a decreased capacity to build and maintain peat soils. Our results show that sawgrass phenotypes in peat vs. marl can differentiate within a single year. If restoration can supply the deeper, more sustained hydrologic conditions that prevent peat oxidation, sawgrass plants will produce organic matter to rebuild peat. The reduced growth of marl sawgrass plants, however, suggests that this will be a slow process. Once sufficient peat has accumulated, increased sawgrass growth will sequester more CO_2_, helping to combat global climate changes.

## CONCLUSIONS

Sawgrass plants grown from seeds collected in a single population diverged morphologically when planted in marl vs. peat soils. The differences in morphology began to develop within 1 mo after transfer to the different soil types, increased over time, and were correlated with soil TP. The changes that resulted resembled the differences seen between sawgrass plants growing in marl vs. peat habitats in the Everglades. This rapid divergence in phenotype supports the hypothesis that marl prairies could be a recent, novel habitat in southern Florida that developed as a result of landscape‐scale draining and burning of Everglades marshes (McVoy et al., 2011).

## AUTHOR CONTRIBUTIONS

J.H.R. performed the growth experiments, oversaw the harvest, did the morphological and growth analyses, and wrote the first draft of the paper. P.C.O. measured photosynthesis, analyzed the photosynthesis data, and wrote the first draft of the photosynthesis results. J.H.R. and P.C.O. collaborated on rewriting and refining the manuscript.

## Supporting information


**APPENDIX S1.** Images of field‐ and mesocosm‐grown sawgrass plants.

**APPENDIX S2.** Mesocosm environment.

**APPENDIX S3.** Mean morphological variables for mesocosm‐grown sawgrass in peat and marl soils.

## Data Availability

Data are available upon request from J. H. Richards.
