# Supplementary material for: A common‐mesocosm experiment recreates sawgrass (Cladium jamaicense) phenotypes from Everglades marl prairies and peat marshes
Source: Am J Bot. 2019 Dec 31;107(1):56–65. doi: 10.1002/ajb2.1411 (PMC7004165; doi:10.1002/ajb2.1411)
Supplement: Supplementary file 1 — APPENDIX S1. Images of field‐ and mesocosm‐grown sawgrass plants. [file AJB2-107-56-s001.docx]

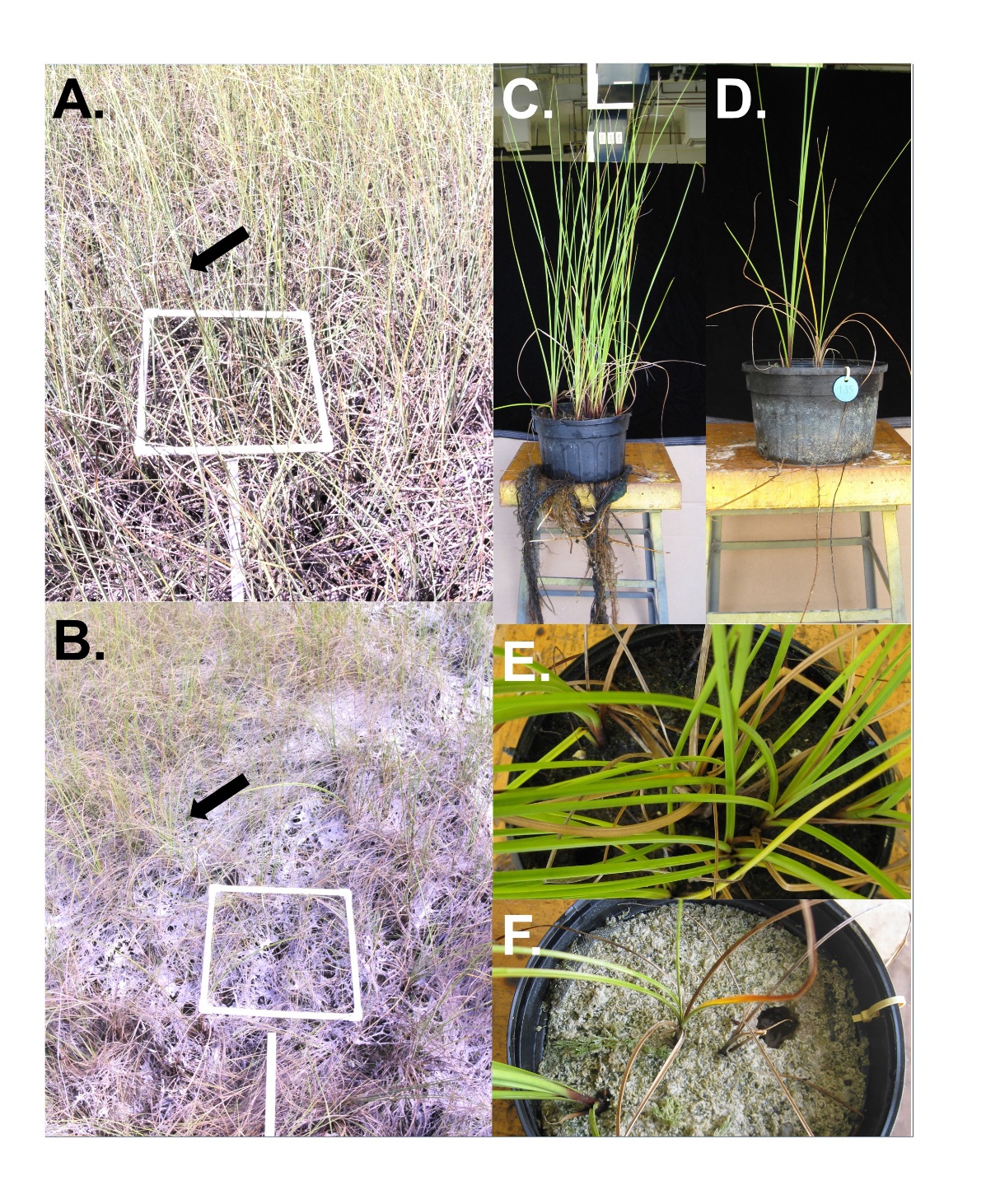
**Appendix S1.** Images of field- and mesocosm-grown sawgrass plants. **A-B.** Sawgrass marsh (A.) and marl prairie (B.) in Everglades National Park, spring 2019. Plants in A. are primarily sawgrass, while plant in B. are sawgrass, other graminods, and herbs with dried periphyton between plants. Quadrat = 0.25 m^2^; arrows point to individual sawgrass culms. C-F. Sawgrass plant grown for 14 mo. in mesocosms in native peat (C., E.) and marl (D., E.) soils. Pots are 20 cm d. Soil core hole visible in F.
