# Supplementary material for: A common‐mesocosm experiment recreates sawgrass (Cladium jamaicense) phenotypes from Everglades marl prairies and peat marshes
Source: Am J Bot. 2019 Dec 31;107(1):56–65. doi: 10.1002/ajb2.1411 (PMC7004165; doi:10.1002/ajb2.1411)
Supplement: Supplementary file 2 — APPENDIX S2. Mesocosm environment. [file AJB2-107-56-s002.docx]

***Appendix S2.*** Mesocosm environment***—***The southern Florida climate is seasonally hot and wet from May through October and cooler and drier from November through April. Over the period of the experiment, day/night temperatures for the hot, wet season averaged 30.9 ± 3.1ºC / 27.2 ± 2.4ºC, while temperatures for the cool, dry season averaged 22.7 ± 3.6ºC / 22.4 ± 3.1ºC. Mesocosm water nutrients were low. Soluble reactive phosphorus was below detection limits in both mesocosms, while total nitrogen was 37.6 ± 9.2 mg L^-1^ in one mesocosm and 117.0 ± 86.9 mg L^-1^ in the other. In both mesocosms, 97% of the nitrogen was ammonium.
