# Supplementary material for: A common‐mesocosm experiment recreates sawgrass (Cladium jamaicense) phenotypes from Everglades marl prairies and peat marshes
Source: Am J Bot. 2019 Dec 31;107(1):56–65. doi: 10.1002/ajb2.1411 (PMC7004165; doi:10.1002/ajb2.1411)
Supplement: Supplementary file 3 — APPENDIX S3. Mean morphological variables for mesocosm‐grown sawgrass in peat and marl soils. [file AJB2-107-56-s003.docx]

***Appendix S3.*** Mean morphological variables for mesocosm-grown sawgrass in peat and marl soils. Mean ± standard error for *C. jamaicense* leaf length and width of the most recently matured leaf (MRM), number (No.) of new and live leaves, and number of branches on plants in marl and peat soils in April after 10.5 months of growth in the mesocosms. N_M_ = 40; N_P_ = 41.

|  | **Marl** | | | **Peat** | | | **Prob.** | **P/M** |
| --- | --- | --- | --- | --- | --- | --- | --- | --- |
| Length MRM (cm) | 32.1 | ± | 1.5 | 52.2 | ± | 1.1 | < 0.01 | 1.63 |
| Width MRM (mm) | 2.4 | ± | 0.1 | 3.3 | ± | 0.2 | < 0.01 | 1.37 |
| No. of new leaves | 3.6 | ± | 0.1 | 3.9 | ± | 0.1 | 0.01 | 1.09 |
| No. of live leaves | 3.0 | ± | 0.2 | 4.3 | ± | 0.2 | < 0.01 | 1.43 |
| No. of branches | 2.3 | ± | 0.1 | 6.6 | ± | 0.6 | < 0.01 | 2.88 |

Notes: MRM = most recently matured leaf; No. = number; Prob. = probability of > χ^2^ in a KW test of differences between plants in marl and peat soils; P/M = ratio of peat to marl for each variable.
